# Supplementary material for: Heparin-based hydrogel scaffolding alters the transcriptomic profile and increases the chemoresistance of MDA-MB-231 triple-negative breast cancer cells
Source: Biomater Sci. 2020 Feb 13;8(10):2786–96. doi: 10.1039/c9bm01481k (PMC7497406; doi:10.1039/c9bm01481k)
Supplement: Supplementary file 2 [file BM-008-C9BM01481K-s002.zip › Supplementary File 4/HP-B hydrogel + EGF (s)vControl/Pathways/my_analysis.Gsea.1545201170987/HALLMARK_CHOLESTEROL_HOMEOSTASIS.html]

Details for gene set HALLMARK\_CHOLESTEROL\_HOMEOSTASIS[GSEA]

|  || Dataset | expr.class.cls#HYDEGF\_versus\_CONTROL.class.cls#HYDEGF\_versus\_CONTROL\_repos |
| Phenotype | class.cls#HYDEGF\_versus\_CONTROL\_repos |
| Upregulated in class | CONTROL |
| GeneSet | HALLMARK\_CHOLESTEROL\_HOMEOSTASIS |
| Enrichment Score (ES) | -0.43564913 |
| Normalized Enrichment Score (NES) | -1.7580246 |
| Nominal p-value | 0.0024038462 |
| FDR q-value | 0.0018882774 |
| FWER p-Value | 0.021 |
Table: GSEA Results Summary

  

Fig 1: Enrichment plot: HALLMARK\_CHOLESTEROL\_HOMEOSTASIS      
 Profile of the Running ES Score & Positions of GeneSet Members on the Rank Ordered List

  

| PROBE | DESCRIPTION (from dataset) | GENE SYMBOL | GENE\_TITLE | RANK IN GENE LIST | RANK METRIC SCORE | RUNNING ES | CORE ENRICHMENT || 1 | ATF3 | na |  |  | 473 | 2.154 | 0.0108 | No |
| 2 | CHKA | na |  |  | 840 | 1.788 | 0.0212 | No |
| 3 | TNFRSF12A | na |  |  | 1130 | 1.611 | 0.0327 | No |
| 4 | PCYT2 | na |  |  | 1481 | 1.439 | 0.0382 | No |
| 5 | EBP | na |  |  | 2627 | 1.097 | -0.0036 | No |
| 6 | ATF5 | na |  |  | 3511 | 0.903 | -0.0348 | No |
| 7 | FASN | na |  |  | 3578 | 0.888 | -0.0236 | No |
| 8 | ATXN2 | na |  |  | 3595 | 0.885 | -0.0098 | No |
| 9 | LSS | na |  |  | 3660 | 0.873 | 0.0012 | No |
| 10 | ETHE1 | na |  |  | 5107 | 0.636 | -0.0639 | No |
| 11 | TRIB3 | na |  |  | 5408 | 0.590 | -0.0698 | No |
| 12 | MVK | na |  |  | 6089 | 0.491 | -0.0973 | No |
| 13 | SEMA3B | na |  |  | 6755 | 0.403 | -0.1254 | No |
| 14 | MVD | na |  |  | 6948 | 0.379 | -0.1291 | No |
| 15 | ABCA2 | na |  |  | 6951 | 0.379 | -0.1230 | No |
| 16 | ALCAM | na |  |  | 7468 | 0.309 | -0.1449 | No |
| 17 | STX5 | na |  |  | 7932 | 0.252 | -0.1649 | No |
| 18 | GNAI1 | na |  |  | 7972 | 0.246 | -0.1629 | No |
| 19 | LDLR | na |  |  | 8020 | 0.241 | -0.1614 | No |
| 20 | FBXO6 | na |  |  | 9760 | 0.042 | -0.2516 | No |
| 21 | ACSS2 | na |  |  | 10065 | 0.010 | -0.2673 | No |
| 22 | FAM129A | na |  |  | 10690 | -0.064 | -0.2989 | No |
| 23 | PMVK | na |  |  | 10851 | -0.085 | -0.3058 | No |
| 24 | GUSB | na |  |  | 10994 | -0.105 | -0.3115 | No |
| 25 | ACAT2 | na |  |  | 11541 | -0.166 | -0.3373 | No |
| 26 | GSTM2 | na |  |  | 11557 | -0.169 | -0.3353 | No |
| 27 | LPL | na |  |  | 11671 | -0.184 | -0.3382 | No |
| 28 | CYP51A1 | na |  |  | 11845 | -0.211 | -0.3437 | No |
| 29 | HMGCS1 | na |  |  | 11862 | -0.213 | -0.3411 | No |
| 30 | TMEM97 | na |  |  | 12724 | -0.318 | -0.3808 | No |
| 31 | GPX8 | na |  |  | 13049 | -0.349 | -0.3920 | No |
| 32 | HSD17B7 | na |  |  | 13211 | -0.372 | -0.3942 | No |
| 33 | TM7SF2 | na |  |  | 13337 | -0.393 | -0.3943 | No |
| 34 | FDPS | na |  |  | 13646 | -0.433 | -0.4032 | No |
| 35 | SREBF2 | na |  |  | 13672 | -0.437 | -0.3973 | No |
| 36 | LGMN | na |  |  | 13973 | -0.484 | -0.4050 | No |
| 37 | PLAUR | na |  |  | 14166 | -0.503 | -0.4068 | No |
| 38 | ECH1 | na |  |  | 14216 | -0.507 | -0.4010 | No |
| 39 | PLSCR1 | na |  |  | 14338 | -0.526 | -0.3986 | No |
| 40 | HMGCR | na |  |  | 14351 | -0.528 | -0.3905 | No |
| 41 | CXCL16 | na |  |  | 15018 | -0.636 | -0.4148 | No |
| 42 | FABP5 | na |  |  | 15412 | -0.703 | -0.4237 | Yes |
| 43 | PPARG | na |  |  | 15629 | -0.748 | -0.4227 | Yes |
| 44 | NFIL3 | na |  |  | 15878 | -0.797 | -0.4225 | Yes |
| 45 | NSDHL | na |  |  | 16044 | -0.831 | -0.4174 | Yes |
| 46 | FDFT1 | na |  |  | 16053 | -0.834 | -0.4041 | Yes |
| 47 | CPEB2 | na |  |  | 16059 | -0.836 | -0.3905 | Yes |
| 48 | JAG1 | na |  |  | 16098 | -0.843 | -0.3786 | Yes |
| 49 | FADS2 | na |  |  | 16193 | -0.868 | -0.3692 | Yes |
| 50 | DHCR7 | na |  |  | 16459 | -0.938 | -0.3676 | Yes |
| 51 | ANTXR2 | na |  |  | 16566 | -0.960 | -0.3572 | Yes |
| 52 | SQLE | na |  |  | 16592 | -0.967 | -0.3426 | Yes |
| 53 | PNRC1 | na |  |  | 16832 | -1.038 | -0.3380 | Yes |
| 54 | STARD4 | na |  |  | 16981 | -1.083 | -0.3278 | Yes |
| 55 | CTNNB1 | na |  |  | 17952 | -1.465 | -0.3543 | Yes |
| 56 | IDI1 | na |  |  | 18098 | -1.547 | -0.3364 | Yes |
| 57 | ANXA5 | na |  |  | 18348 | -1.710 | -0.3212 | Yes |
| 58 | S100A11 | na |  |  | 18360 | -1.721 | -0.2933 | Yes |
| 59 | LGALS3 | na |  |  | 18514 | -1.848 | -0.2708 | Yes |
| 60 | CLU | na |  |  | 18586 | -1.909 | -0.2431 | Yes |
| 61 | TP53INP1 | na |  |  | 18743 | -2.103 | -0.2165 | Yes |
| 62 | MAL2 | na |  |  | 18756 | -2.121 | -0.1821 | Yes |
| 63 | PDK3 | na |  |  | 18854 | -2.278 | -0.1496 | Yes |
| 64 | SCD | na |  |  | 18875 | -2.317 | -0.1124 | Yes |
| 65 | ERRFI1 | na |  |  | 18921 | -2.402 | -0.0751 | Yes |
| 66 | CD9 | na |  |  | 19017 | -2.631 | -0.0366 | Yes |
| 67 | ALDOC | na |  |  | 19062 | -2.788 | 0.0071 | Yes |
Table: GSEA details [plain text format]

  

Fig 2: HALLMARK\_CHOLESTEROL\_HOMEOSTASIS      
 Blue-Pink O' Gram in the Space of the Analyzed GeneSet

  

Fig 3: HALLMARK\_CHOLESTEROL\_HOMEOSTASIS: Random ES distribution      
 Gene set null distribution of ES for **HALLMARK\_CHOLESTEROL\_HOMEOSTASIS**
